# Depression, anxiety, and sleep problems among Chinese university students enrolled in basketball elective courses: a network psychometric analysis

**DOI:** 10.3389/fpsyg.2026.1738600

**Published:** 2026-03-04

**Authors:** Xinle Wu, Xiaofeng Wang

**Affiliations:** 1 Jilin University, Changchun, Jilin Province, China; 2 Physical Education College of Jilin University, Changchun, Jilin Province, China; 3 Northeast Asian Research Center of Jilin University, Changchun, Jilin Province, China

**Keywords:** anxiety, basketball, depression, mental health, network analysis, sleep problems, university students

## Abstract

**Background:**

University students face a substantial mental health burden. Although team sports such as basketball are recognized for their benefits, the symptom-level mechanisms linking depression, anxiety, and sleep disorders within this physically active population remain unclear.

**Methods:**

A cross-sectional study was conducted with 2,108 Chinese university students enrolled in basketball elective courses at Jilin University in Changchun province, China. Network analysis was applied to construct symptom networks of depression (PHQ-9), anxiety (GAD-7), and sleep problems (PSQI). Centrality metrics (strength, bridge strength) identified core symptoms and connectors between symptom clusters. Network stability was assessed via bootstrap procedures, and gender differences were examined using Network Comparison Tests. A sensitivity analysis was performed by reconstructing networks after excluding the PHQ3 to assess their influence on network structure.

**Results:**

Prevalence rates were 16.08% for anxiety, 19.83% for depression, and 23.34% for sleep problems. In the depression-anxiety network, uncontrollable worry (GAD2) and irritability (GAD6) emerged as core symptoms, while restlessness (GAD5) and irritability (GAD6) functioned as key bridge symptoms. In the tri-domain network incorporating sleep problems, daytime dysfunction (PSQI7) exhibited the highest centrality and bridge strength, linking sleep disturbances to emotional symptoms. Gender comparisons revealed no significant differences in network structure or global strength. The sensitivity analysis confirmed that the overall network topology and key bridge symptoms remained stable after excluding PHQ3.

**Conclusion:**

Depression, anxiety, and sleep problems form interconnected symptom networks in basketball elective students, with specific symptoms acting as critical nodes and bridges. Targeted interventions focusing on daytime dysfunction, irritability, and uncontrollable worry may disrupt symptom propagation and improve mental health outcomes in this population.

## 1 Introduction

Higher education students represent a high-risk population for mental health disorders (Auerbach et al., 2018). According to the World Mental Health International College Student (WMH-ICS) initiative, approximately 35.3% of students globally have screened positive for at least one common DSM-IV anxiety, mood, or substance use disorder in their lifetime, with 31.4% reporting symptoms in the past year (Auerbach et al., 2018). Meta-analyses reveal a substantial mental health burden among Chinese university students (Deng et al., 2021; Lin et al., 2018, Lin et al., 2025), with depression prevalence at 34.70% (Lin et al., 2025), anxiety disorders at 32% during the COVID-19 pandemic (Deng et al., 2021), and sleep disorders at 25.7% (Li et al., 2018). The GBD 2021 study further reveals a rising trend in the burden of depression and anxiety in China over the past 3 decades (Tian et al., 2021). These mental health issues not only impair academic performance and quality of life but are also associated with increased risks of suicidal ideation and related high-risk behaviors (Zhou et al., 2021).

The growing prevalence of mental health issues among university students worldwide has made this topic a key concern intersecting education, public health, and sports science. In recent years, physical activity is increasingly recognized as a potential strategy for promoting mental health (Huang et al., 2024; Smith and Merwin, 2021; Watson et al., 2017). Global initiatives, including the WHO's Global Action Plan on Physical Activity 2018–2030: More Activity, Better Health (World Health Organization, 2018) and the WHO Guidelines on Physical Activity and Sedentary Behavior (World Health Organization, 2020), emphasize the benefits of physical activity for mental health. Meta-analyses consistently support these findings, demonstrating that physical activity can alleviate symptoms of depression and anxiety (Heissel et al., 2023; Pearce et al., 2022; Recchia et al., 2023). Among various physical activities, team sports (such as basketball) demonstrate unique psychological and social benefits. Beyond enhancing physical endurance and supporting cardiovascular and musculoskeletal health, they foster social interaction and well being, thereby contributing to mental health (Castagna et al., 2018). A 12-week quasi-experimental study involving Chinese university students further confirmed that team sports can help reduce depressive symptoms and improve sleep quality (Johnston et al., 2021). Moreover, compared to individual exercise, team sports may promote greater physical and mental health benefits and encourage long-term adherence (Andersen et al., 2019). Basketball is a widely offered team sport in university physical education programs, distinguished by its competitive, interactive, and cooperative features. The course design typically incorporates structured activities, including skill drills, teamwork exercises, and competitive scenarios. Thus, students enrolled in these courses are simultaneously influenced by multiple psychosocial and physiological factors, such as physical exertion, social support, and performance pressure. This group not only experiences psychological stresses common to university students but also operates within a distinct environment shaped by sports pedagogy and team dynamics. Therefore, the presentation, mechanisms, and interactions of emotional and sleep problems in this population may differ from those in the general student body or among students who participate only in individual sports.

Multiple comparative studies have demonstrated that students who participate in team sports experience notable psychological health benefits. Relative to their non-athlete peers, college student-athletes exhibit significantly lower rates of anxiety, eating, mood, sleep, and other disorders (Edwards and Froehle, 2023). Longitudinal research further indicates a strong association between sustained involvement in team sports and reduced depression levels in early adulthood (Sabiston et al., 2016). Drawing on the “Conceptual Model of Health through Sport,” Caviedes et al. highlighted that, compared with general physical activity, team sports provide additional mental health advantages due to their distinct social characteristics (Eime et al., 2013). Mediation analyses confirm that social support plays a crucial role in the mechanism through which sports participation alleviates anxiety among university students, implying that the social bonds developed in team settings may indirectly lower anxiety (Liu and Shi, 2023). Although direct comparisons between basketball players and other student groups remain limited, the structured, cooperative, and goal-oriented nature of basketball suggests that its mental-health-promoting effects could be especially pronounced.

Although numerous studies have confirmed a positive association between physical exercise and mental health, systematic investigations into the specific effects of ball game elective courses among university students remain scarce. Particularly lacking are in-depth analyses of the internal structure of psychological symptoms and their interaction mechanisms. Furthermore, existing research predominantly employs traditional variable-centered statistical methods, such as regression analysis (Alnawwar et al., 2023; Rezaie et al., 2023) or structural equation modeling (Liu et al., 2023; Zhao et al., 2025). While these approaches can reveal overall variable-level trends, they are limited in capturing the dynamic interplay of symptoms within individuals. Additionally, most studies tend to treat depression, anxiety, and sleep disturbances as independent dimensions (Alnawwar et al., 2023; Liu et al., 2023; Rezaie et al., 2023; Zhao et al., 2025), overlooking their frequently co-occurring and mutually reinforcing nature in real-world contexts. This oversight constrains a comprehensive understanding of the complex mechanisms underlying the development of these mental disorders.

In recent years, the emergence of network psychometrics has provided a new research path for understanding mental health disorders (Borsboom, 2017). A growing body of research suggests that depression, anxiety, and sleep disturbances do not occur in isolation. Instead, they form a cluster of mutually reinforcing symptoms, interconnected through key clinical features that create bidirectional loops and cascading pathways (Borsboom, 2017; Epskamp et al., 2018; Jones et al., 2021). In college student populations, multiple studies have found that symptoms such as uncontrollable worry, restlessness, and irritability in the anxiety dimension, fatigue, and loss of interest in the depression dimension, as well as poor sleep quality and daytime dysfunction in the sleep dimension, often occupy central positions in the network or serve as bridges across communities (Bai et al., 2021; Li et al., 2024; Tao et al., 2022). Moreover, network structure and key bridge symptoms may vary across subgroups based on stress levels or gender, underscoring the need for precise, and context-specific interventions (Li et al., 2024; Tao et al., 2022).

Therefore, this study employs network analysis to construct symptom networks for depression, anxiety, and sleep quality among Chinese university students enrolled in basketball elective courses at Jilin University in Changchun province, to provide actionable evidence for integrated psychological and physical intervention strategies in higher education.

## 2 Materials and methods

### 2.1 Study design

#### 2.1.1 Participants and procedures

This study ultimately included a sample of 2,108 participants from Jilin University in Changchun province, China. Employing a cross-sectional design, the research was conducted at Jilin University from September to October 2023 using cluster-stratified convenience sampling. The study population comprised all students enrolled in the university's basketball elective classes (total *N* = 2,568). The specific steps were as follows: Firstly, all basketball elective classes published by the Public Sports Department of Jilin University were designated as primary sampling units (clusters). Classes were included in their entirety to avoid splitting participants across classes and minimize disruption to physical education teaching. The stratification factor was year level (1st-year/2nd-year) to account for differences in academic pressure. With prior informed consent from instructors, electronic questionnaire QR codes were distributed during the 2nd week of classes and collected immediately, ensuring a response rate exceeding 95%. Absentees or refusals were recorded as “non-respondents” without replacement sampling, adhering to ethical and teaching arrangements. Data collection utilized an electronic questionnaire covering depression (PHQ-9), anxiety (GAD-7), sleep quality (Pittsburgh Sleep Quality Index, PSQI), and demographic information. Distribution via classroom sessions ensured high response rates and data quality. The sample size was determined through network power simulation analysis (Epskamp and Fried, 2018; Supplementary Figure S1), where the estimated correlation coefficient for intensity centrality decayed ≤ 0.05 when *n* ≥ 1,800, hence a target sample of 2,000 participants was set. A total of 2,245 responses were ultimately collected. After excluding systematic responses and those with >20% missing data, 2,108 valid samples remained, meeting the stability criterion of CS coefficient ≥0.75.

This research protocol has been approved by the Ethics Review Committee of Jilin University. All participants read and signed an electronic informed consent form prior to completing the questionnaire, and were informed of the voluntary and anonymous nature of their participation.

### 2.2 Measurements

This study employed the following measurement instruments to assess participants' symptoms of depression, anxiety, and sleep problems. Supplementary Table S1 provides detailed descriptions of the subscale items for each instrument.

#### 2.2.1 Anxiety symptoms

Anxiety symptoms are evaluated using the Generalized Anxiety Disorder 7-Item Scale (GAD-7; Spitzer et al., 2006). The GAD-7 consists of seven items measuring anxiety symptoms over the previous 2 weeks. Each item is rated on a scale from 0 to 3, where 0 represents “not at all” and 3 represents “nearly every day.” Higher total scores indicate greater anxiety severity. The GAD-7 has demonstrated strong reliability and validity in previous studies, with a Cronbach's alpha of 0.92 indicating excellent internal consistency (Spitzer et al., 2006). Anxiety levels were classified based on GAD-7 total scores: 0–4 = minimal, 5–9 = mild, 10–14 = moderate, and 15–21 = severe (Spitzer et al., 2006). A GAD-7 score of 10 or higher is considered clinically significant (Löwe et al., 2008).

#### 2.2.2 Depression symptoms

Depressive symptoms are assessed using the Patient Health Questionnaire-9 (PHQ-9; Spitzer et al., 1999). The PHQ-9 is a widely validated self-report instrument comprising nine items that evaluate depressive symptom severity over the preceding 2 weeks. Each item is scored from 0 to 3, with higher total scores reflecting more severe depression. The PHQ-9 exhibits strong psychometric properties, including a Cronbach's alpha of 0.851 (Maroufizadeh et al., 2019), which indicates good internal consistency. The PHQ-9 has been extensively validated within Chinese populations, establishing it as a reliable instrument for assessing depressive symptoms (Wang et al., 2014). Depression severity was classified according to PHQ-9 scores as follows: 1–4 = minimal, 5–9 = mild, 10–14 = moderate, 15–19 = moderately severe, and 20–27 = severe (Maurer et al., 2018). Accordingly, a cutoff score of 10 was applied to define the presence of depressive symptoms (Kroenke et al., 2001).

#### 2.2.3 Sleep problems

Sleep problems are measured using the Pittsburgh Sleep Quality Index (PSQI; Buysse and Reynolds, 1989). The PSQI includes 18 items that assess seven sleep domains over the past month. Each domain is scored from 0 to 3, with higher scores representing poorer sleep quality. The PSQI has shown excellent reliability and validity, with a Cronbach's alpha of 0.83, indicating good internal consistency (Buysse and Reynolds, 1989). We defined a PSQI score greater than 7 as indicative of sleep disorders (Buysse et al., 1989).

### 2.3 Statistical analysis

All statistical analyses were performed using R-Studio (version 4.3.2). Descriptive statistics summarized the demographic characteristics and scale scores. Categorical variables are presented as frequencies and percentages (*n*, %), and continuous variables as means with standard deviations (SD). We subsequently constructed comorbidity networks for depression, anxiety. Network analysis elucidates the bivariate relationships among multiple variables, revealing the structural and functional characteristics of complex psychopathological systems—such as core components, co-occurrence patterns, and pivotal nodes—which are often not fully captured by traditional regression or latent variable models (McNally, 2021). Prior to network construction, potential item redundancy was assessed using the “goldbricker” function from the “networktools” R package. The “goldbricker” function identifies potentially redundant node pairs by assessing the correlation profiles between each candidate pair and all other nodes in the network. If two nodes exhibit similar correlations with all other nodes (i.e., statistically significant differences in correlations are observed in less than 25% of cases), they are considered redundant nodes (Hittner et al., 2003). The robustness of the network was confirmed through stability analysis. The significance threshold for all tests was set at *P* < 0.05.

#### 2.3.1 Network construction

Network analysis was conducted to elucidate the complex interrelationships among depression, anxiety, and sleep problems, and to identify core and bridge symptoms within this network. The network structure was estimated using a Graphical Gaussian Model (GGM; Epskamp et al., 2018). To limit spurious connections and obtain a sparse network, the Least Absolute Shrinkage and Selection Operator (LASSO) regularization was applied. Model selection was governed by minimizing the Extended Bayesian Information Criterion (EBIC; Friedman et al., 2008). This approach shrinks trivial edge weights to zero, thereby simplifying the network structure. In this model, the gamma hyperparameter was set to a default value of 0.5 (Epskamp and Fried, 2018). To identify unique direct associations between syndemic indicators, we utilized partial polychoric correlations (Borsboom and Cramer, 2013). By statistically accounting for the influence of all other network nodes, this method isolates the specific relationship between any given pair of variables, eliminating spurious connections (Borsboom and Cramer, 2013).

The network structure was estimated using the R package “qgraph” (Epskamp et al., 2012). Each node represents a symptom, and edges represent partial correlations between pairs of symptoms. Solid blue edges depict positive associations, while dashed red edges indicate negative associations; the thickness of an edge corresponds to the strength of the relationship (Epskamp et al., 2012).

#### 2.3.2 Centrality measures

Node strength centrality and bridge strength centrality were computed to quantify the importance and connectivity of each node within the network (Opsahl et al., 2010). Strength centrality represents the absolute sum of a node's direct connections, reflecting its overall connectivity. Bridge strength centrality specifically measures a node's capacity to connect different symptom clusters or communities (Jones et al., 2021).

#### 2.3.3 Network stability and accuracy

The stability and accuracy of the estimated network were evaluated using the R package “bootnet” (Epskamp et al., 2018). The correlation stability coefficient (CS-C) and 95% bootstrap confidence intervals (CIs) were computed to assess the robustness of the network structure and the precision of edge weight estimates. A non-parametric bootstrap procedure was used to examine the stability of edge weights; narrower 95% CIs indicate greater estimation precision. Typically, a CS-C value above 0.25 is generally considered acceptable, while a value exceeding 0.50 indicates good stability (Epskamp et al., 2018).

#### 2.3.4 Network comparison

Network Comparison Tests (NCT) were performed to examine potential differences in network structure between gender subgroups. These tests evaluated overall network structure (invariance), global strength (the sum of absolute edge weights), and specific edge weights. Permutation-based tests (1,000 permutations) were employed to statistically assess differences in global strength and network structure (i.e., the distribution of edge weights) between the subgroups (van Borkulo et al., 2023).

#### 2.3.5 Sensitivity analysis

To evaluate the potential influence of the PHQ3 items (Sleep Dysregulation) on symptom network analysis, we performed a sensitivity analysis. Specifically, after removing the PHQ3, we reconstructed the networks for depression (PHQ-9), anxiety (GAD-7), and sleep problems (PSQI). The overall network structure was then compared before and after the exclusion of PHQ3.

## 3 Results

### 3.1 Participant characteristics

The baseline and demographic characteristics of the participants are presented in Table 1. The study included 2,108 college students enrolled in a basketball elective course, with a mean age of 19.18 years (SD = 1.09). The sample comprised 1,359 males (64.47%) and 749 females (35.53%). Psychometric assessments revealed mean scores of 5.71 (SD = 4.25) on the GAD-7 and 5.64 (SD = 7.06) on the PHQ-9. Based on established cutoffs, 16.08% (*n* = 339) of participants exhibited anxiety symptoms, while 19.83% (*n* = 418) displayed depressive symptoms. The mean PSQI score was 5.11 (SD = 3.67), with 23.34% (*n* = 492) experiencing sleep problems. Supplementary Table S2 details the distribution, network centrality, and predictability of nodes for the GAD-7, PHQ-9, and PSQI items.

**Table 1:** Baseline and demographic characteristics of the study sample (*n* = 2,108).

**Variables**	**Mean/*N***	**SD/%**
Age (year)	19.18	1.09
Height (m)	1.75	0.10
Weight (kg)	71.05	17.29
BMI (kg/m^2^)	23.18	4.89
**Gender**		
Male	1,359	64.47%
Female	749	35.53%
**Residence**		
Urban	805	38.19%
Rural	1,303	61.81%
**Family sibling status**		
Only child	1,093	51.85%
Non-only child	1,015	48.15%
**Speciality**		
Humanities and social sciences	788	37.38%
Science and engineering	1,203	57.07%
Arts	117	5.55%
**Smoking**		
Rarely or never	1,602	76.00%
Occasionally	212	10.06%
Frequently or almost every day	294	13.95%
**E-cigarette use**		
Rarely or never	1,935	91.79%
Occasionally	110	5.22%
Frequently or almost every day	63	2.99%
**Alcohol consumption**		
Rarely or never	1,663	78.89%
Occasionally	363	17.22%
Frequently or almost every day	82	3.89%
**Coffee consumption**		
Rarely or never	1,328	63.00%
Occasionally	612	29.03%
Frequently or almost every day	168	7.97%
**Tea consumption**		
Rarely or never	1,277	60.58%
Occasionally	667	31.64%
Frequently or almost every day	164	7.78%
**Short video software viewing time**		
Low usage duration: < 1 h	867	41.13%
Medium usage duration: 1–4 h	858	40.70%
High usage duration:>4 h	383	18.17%
GAD-7	5.71	4.25
**Anxiety**		
No anxiety (0–9)	1,769	83.92%
With anxiety (10–21)	339	16.08%
PHQ-9	5.64	7.06
**Depression**		
No depression (0–9)	1,690	80.17%
With depression (10–27)	418	19.83%
Pittsburgh sleep quality index (PSQI)	5.11	3.67
**Sleep problem**		
Normal sleep (0–7)	1,616	76.66%
Have sleep problems (8–21)	492	23.34%

### 3.2 Networks of depressive and anxiety symptoms

**Figure 1:**
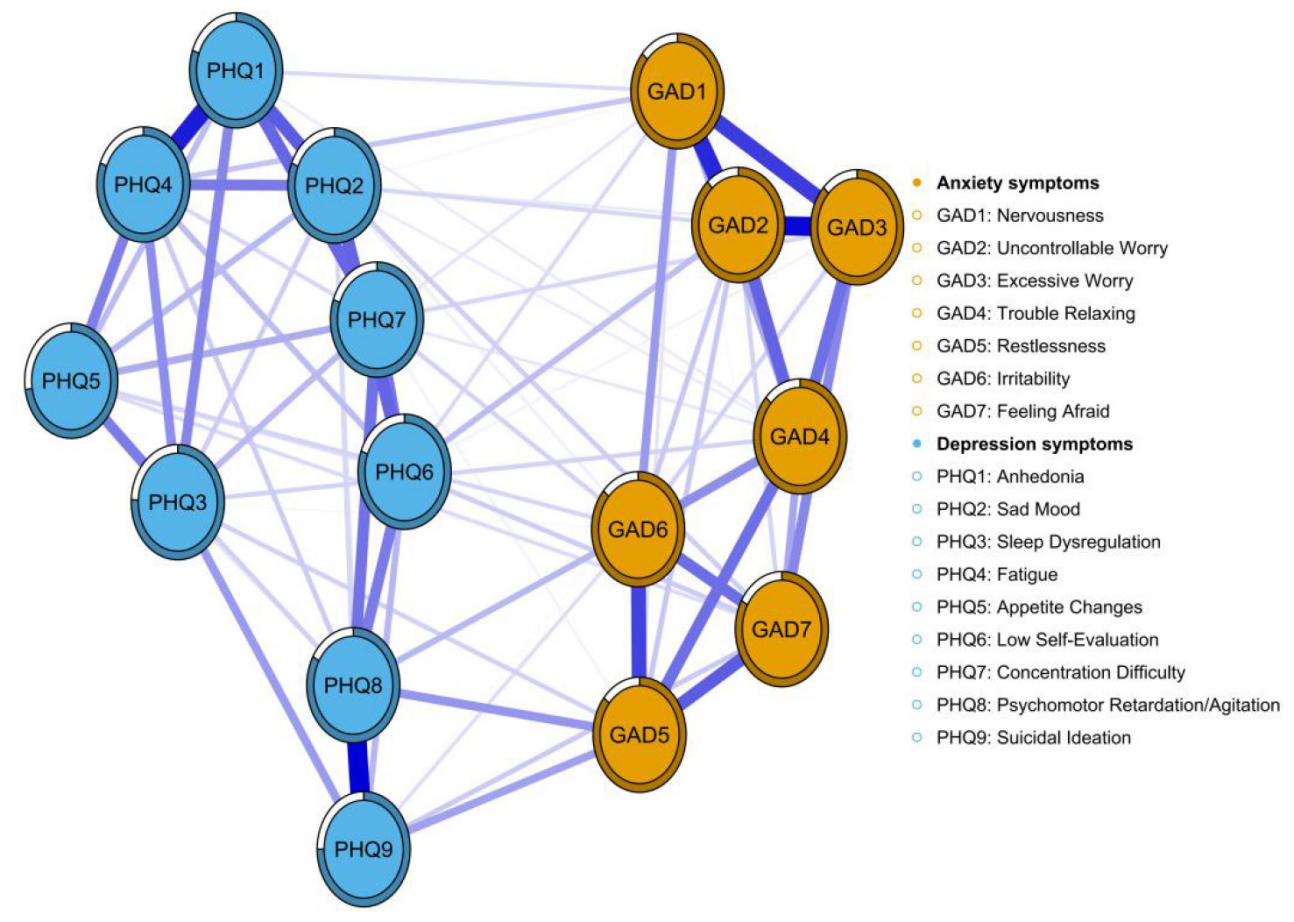
Network analysis of anxiety (GAD-7) and depression (PHQ-9) symptoms among the study participants. Note: Nodes represent individual symptoms; edges denote partial correlations after controlling for all other variables. Blue solid lines indicate positive associations, red dashed lines indicate negative associations; edge thickness is proportional to the strength of the association.

**Figure 2:**
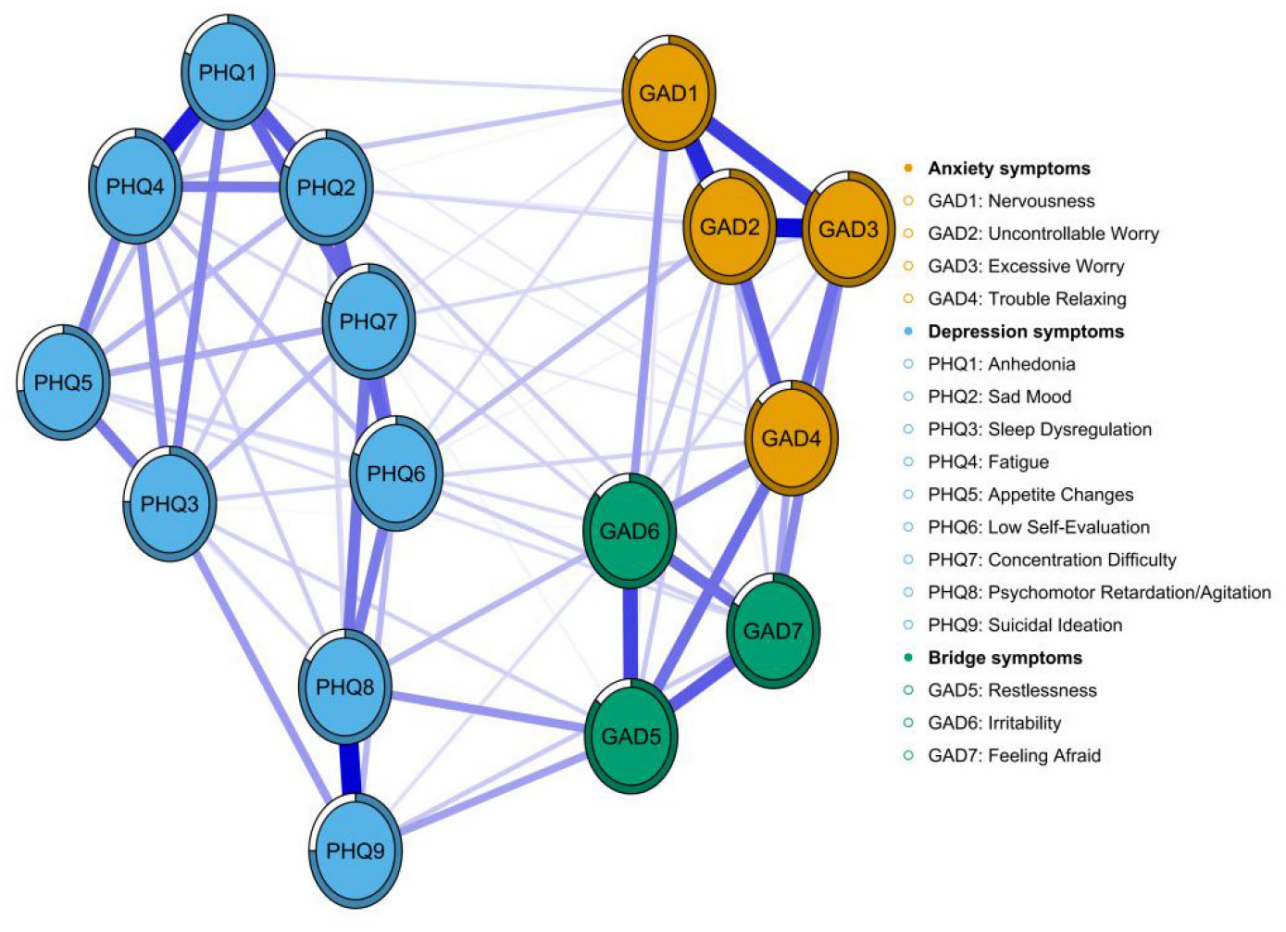
Bridge symptoms within the depression–anxiety symptom network among the study participants. Note: Orange nodes represent GAD-7 anxiety items, blue nodes PHQ-9 depression items, and green nodes bridge symptoms. Edges are regularized partial correlations; blue solid lines indicate positive, red dashed lines negative associations, with thickness proportional to the strength of the association.

Figure 1 depicts the overall network structure of depressive and anxiety symptoms among college students enrolled in basketball courses. The network comprised 82 non-zero edges out of a possible 120, with a density of 0.683 and an average edge weight of 0.065. Strength centrality analysis (Supplementary Figure 2A) identified GAD2 (Uncontrollable Worry), GAD6 (Irritability), and PHQ8 (Psychomotor Retardation/Agitation) as the most central nodes, underscoring their pivotal roles in the depressive-anxiety network. Following LASSO regularized estimation and bridge strength ranking, three anxiety items—GAD5 (Restlessness), GAD6 (Irritability), and GAD7 (Feeling Afraid)—were highlighted in green due to their pronounced cross-cluster connectivity (Figure 2) and high bridge strength centrality (all *z*-values >0.75; Supplementary Figure 2B), confirming their key role in linking anxiety and depression comorbidity. Their direct coupling with core depressive symptoms (PHQ8, PHQ9) significantly reduced the geodesic distance between communities. Among these, GAD5 ranked first in bridging strength (Supplementary Figure 2B) and exhibited strong correlations with both PHQ8 (Psychomotor Retardation/Agitation) and PHQ9 (Suicidal Ideation). This indicates that “Restlessness” (GAD5) serves as the pivotal symptom node linking the two major symptom clusters of anxiety and depression within this network. GAD6 (Irritability) demonstrated strong performance in both centrality measures, preliminarily validating its role as a pivotal bridging node in the depression → anxiety pathway. Systematic stability and diagnostic accuracy analyses confirmed the numerical robustness of depression-anxiety network (Supplementary Figure 3).

### 3.3 Networks of depressive, anxiety, and sleep problem symptoms

**Figure 3:**
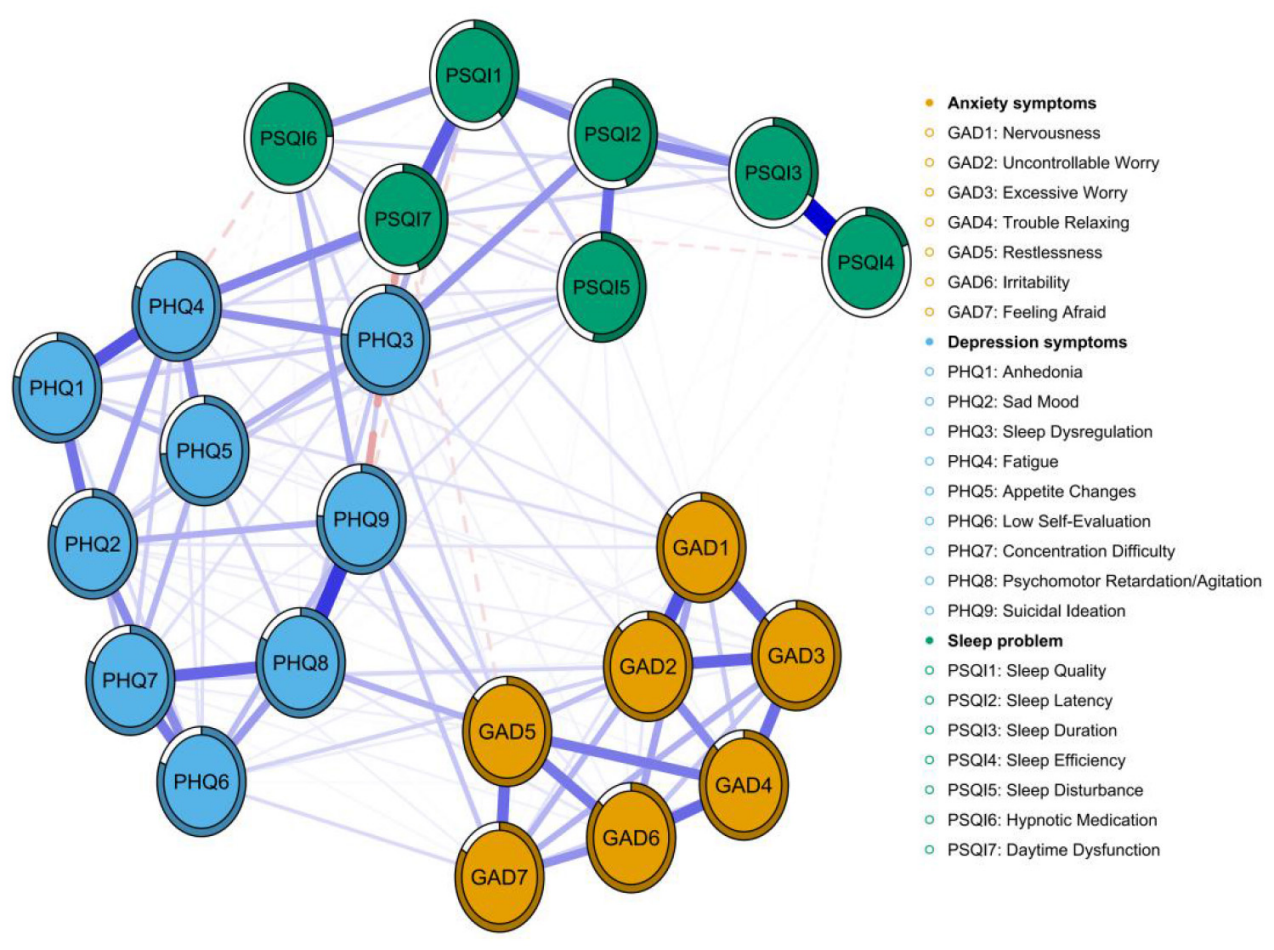
Symptom network of depression (PHQ-9), anxiety (GAD-7) and sleep problems (PSQI) among the study participants. Note: Nodes represent individual symptoms: orange nodes represent GAD-7 anxiety items, blue nodes PHQ-9 depression items, and green nodes PSQI sleep problem items. Edges denote partial correlations after controlling for all other variables. Blue solid lines indicate positive associations, red dashed lines indicate negative associations; edge thickness is proportional to the strength of the association.

Figure 3 depicts the symptom network of depression (PHQ-9), anxiety (GAD-7), and sleep problems (PSQI) among college students enrolled in a basketball course. Of the 253 potential edges, 162 demonstrated significant associations, resulting in a network density of 0.640 and a mean edge weight of 0.041. Among all the edges, the strongest edge was observed between PSQI4 (sleep efficiency) and PSQI3 (sleep duration) (*r* = 0.31), followed by the edge between PHQ8 (psychomotor retardation/agitation) and PHQ9 (suicidal ideation; *r* = 0.31; Supplementary Table S3). Furthermore, the standardized strength centrality (*z*-score) analysis (Supplementary Figure 4, Table S4) identified PSQI7 (daytime dysfunction) and PSQI1 (sleep quality) as the most central symptoms within the entire network, indicating their paramount influence on the co-occurrence of sleep, anxiety, and depression symptoms after controlling for all other nodes. Subsequent bridge strength analysis (Supplementary Figure 5, Table S4) revealed that PSQI7 also exhibited the highest bridge strength, confirming its role as a primary connector across different symptom communities. According to the centrality plot of three-symptom network (Supplementary Figure 6A), PHQ4 (Fatigue), PHQ9 (Suicidal Ideation), and PSQI7 (Daytime Dysfunction) displayed the highest centrality, identifying them as the most influential comorbid symptoms. Collectively, these findings demonstrate that daytime dysfunction serves as the key node through which sleep problems exert spillover effects on emotion symptoms.

**Figure 4:**
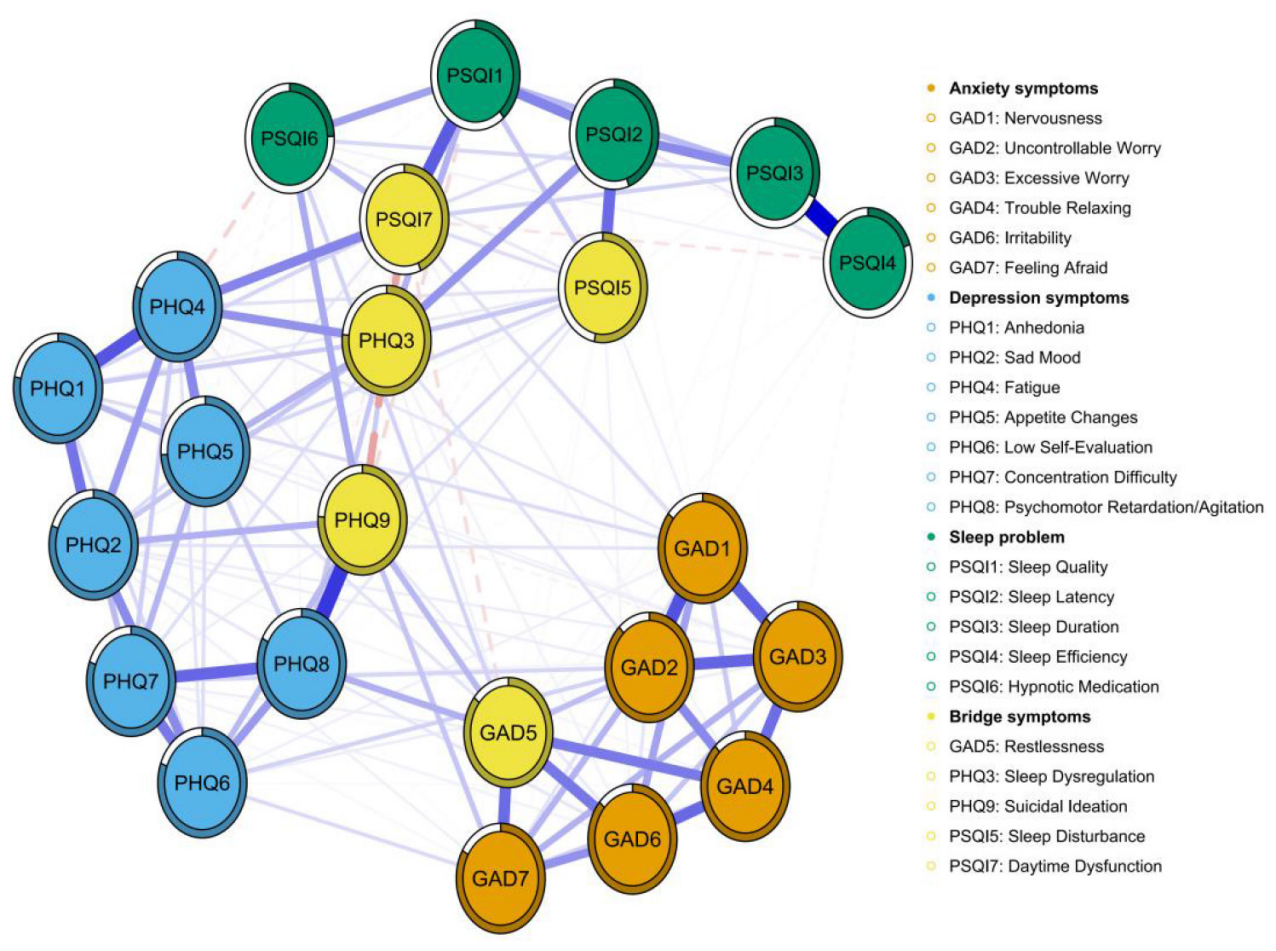
Bridge symptoms within the “depression–anxiety–sleep problem” symptom network among the study participants. Note: Orange nodes represent GAD-7 anxiety items, blue nodes PHQ-9 depression items, green nodes PSQI sleep problem items, and yellow nodes bridge symptoms. Edges are regularized partial correlations; blue solid lines indicate positive, red dashed lines negative associations, with thickness proportional to the strength of the association.

Figure 4 illustrates the bridging symptoms and their associative patterns within the tri-domain symptom network of depression, anxiety, and sleep problems among university students enrolled in basketball courses. Within this network, yellow nodes denote bridging symptoms: GAD5 (Restlessness), representing an anxiety symptom, exhibits a negative correlation with PSQI7 (Daytime Dysfunction) within the sleep problems domain and a positive correlation with PHQ9 (Suicidal Ideation) within the depression domain. PHQ9 (Suicidal Ideation) exhibits a strong negative correlation with PSQI7 (Daytime Dysfunction). PSQI5 (Sleep Disturbance) and PSQI7 (Daytime Dysfunction), as core sleep disorder symptoms, demonstrate multiple associations with both anxiety, and depressive symptoms. Similarly, the bridge strength centrality diagram indicates that PSQI7 (Daytime Dysfunction), PHQ9 (Suicidal Ideation), and PHQ3 (Sleep Dysregulation) exhibit high bridge strength centrality, playing a pivotal role in connecting distinct symptom clusters (Supplementary Figure 6B).

The stability and accuracy of the three-symptom network are presented in Supplementary Figure 7. Bootstrap difference tests (2,000 samples) revealed that most node centrality differences were not statistically significant (Supplementary Figures 7A, B). Case-dropping bootstrap analysis demonstrated that the correlations for both node strength and bridge strength remained high (CS ≥ 0.50) as the subset proportion decreased, indicating robust network stability (Supplementary Figure 7C). Additionally, the narrower 95% confidence intervals of the bootstrap results of edge weights in the network, shown in Supplementary Figure 7D, confirm the precision and replicability of the major edge weight estimates in the network.

### 3.4 Network comparison by gender

**Figure 5:**
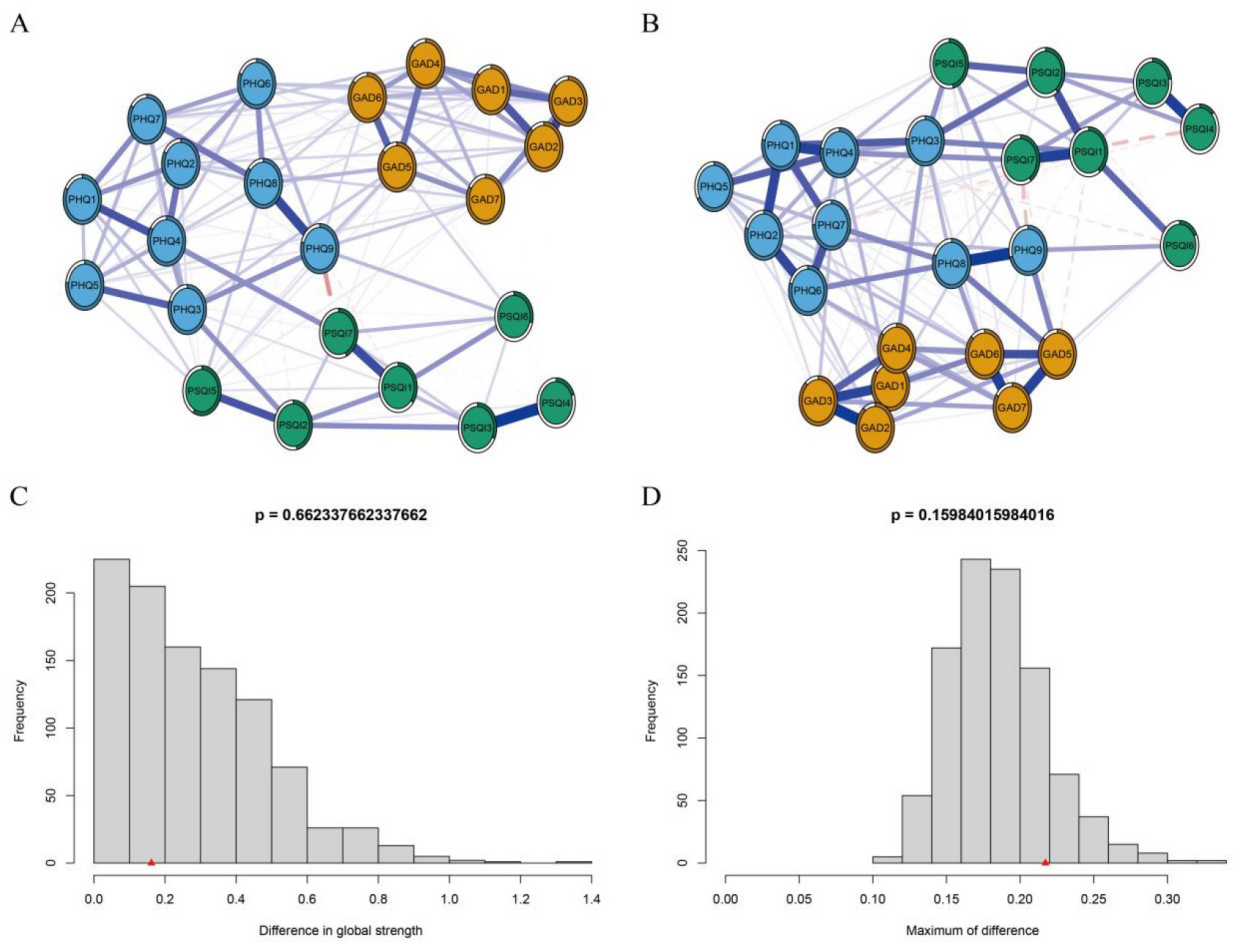
Comparative network analysis of mental health symptoms among the study participants by Gender. **(A)** Network structure for male participants (*n* = 1,359). **(B)** Network structure for female participants (*n* = 749). **(C)** Frequency distribution of differences in global strength between genders, indicating no significant difference (global strength for males: 10.94; for females: 10.78; *P* = 0.662). **(D)** Bootstrap plot of the difference in network structure, showing no significant difference (*M* = 0.217, *P* = 0.160). The stability of edge weights was evaluated using permutation tests, with all Bonferroni-Holm adjusted *P* values exceeding 0.05.

The network comparison test between genders in the college student basketball course revealed no significant differences in global network strength (Figure 5C: 10.94 for males vs. 10.78 for females; *P* = 0.662). Similarly, no significant difference in network structure was observed (Figure 5D: *M* = 0.217, *P* = 0.160). Furthermore, the permutation test of edge weights showed that all *P*-values remained non-significant after Bonferroni-Holm correction (all *P* > 0.05), which means stable network structure.

### 3.5 Sensitivity analysis

**Figure 6:**
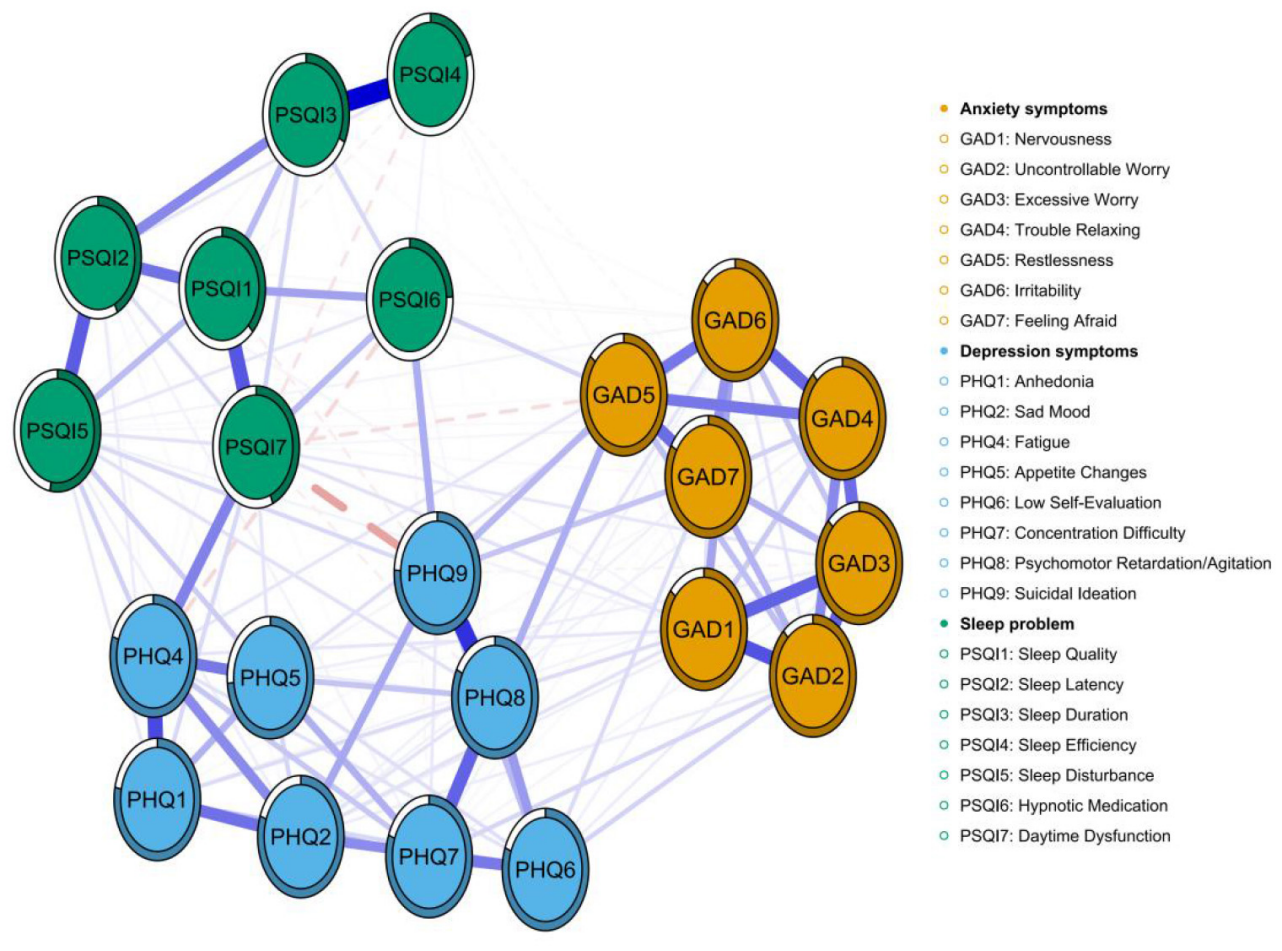
Symptom network of depression (PHQ-9), anxiety (GAD-7), and sleep problems (PSQI) excluding PHQ3 among study participants.

**Figure 7:**
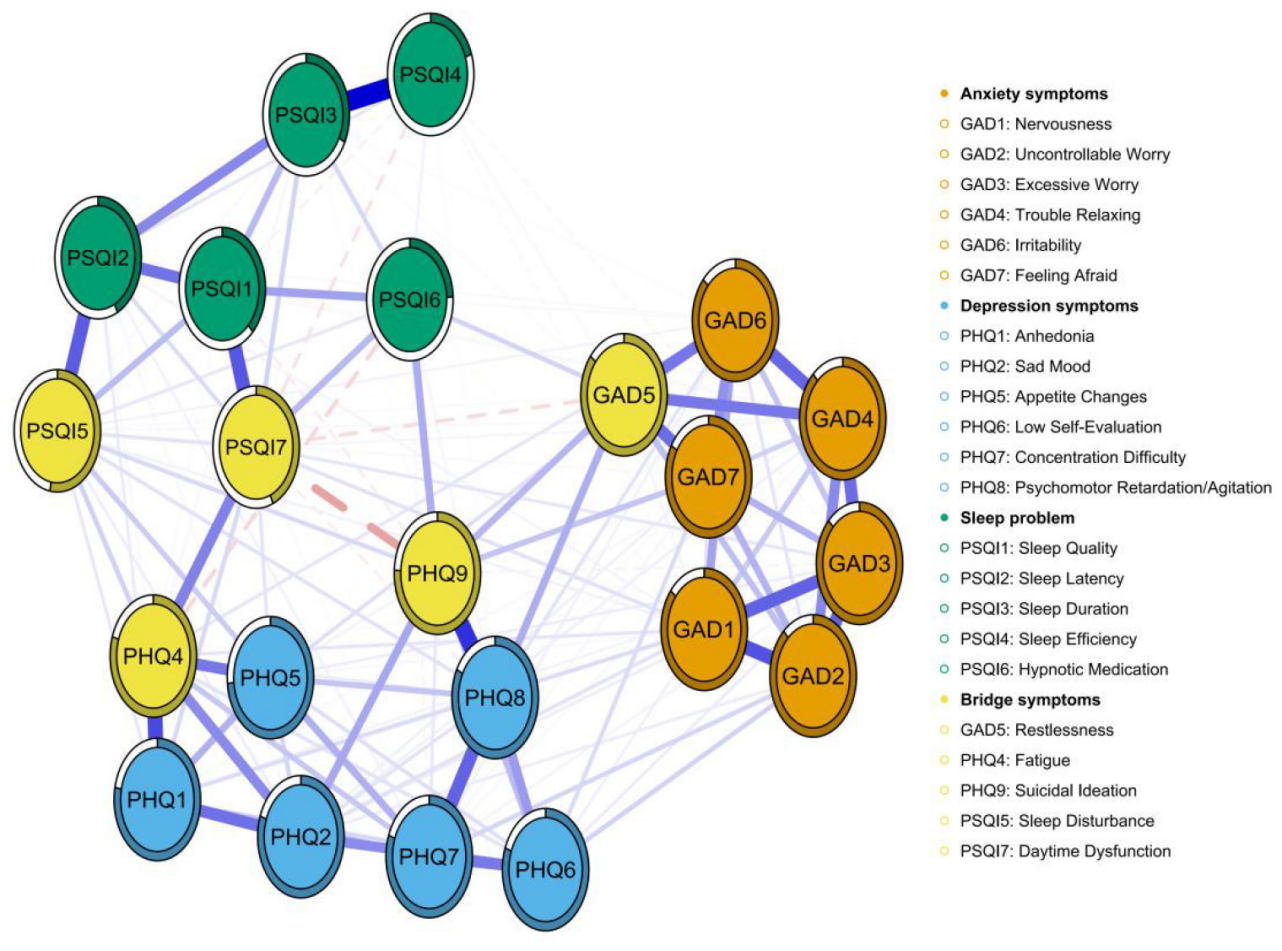
Bridge symptoms within the “depression–anxiety–sleep problem” symptom network excluding PHQ3 among the study participants.

In the sensitivity analysis, the network structure and symptom relationships were examined after the removal of the PHQ3 (Sleep Dysregulation). Following its exclusion, the network retained 22 nodes and 154 non-zero edges, with an average edge weight of 0.0431, indicating generally weak pairwise symptom associations. Although the connection between sleep-related and emotional symptoms weakened, the overall network stability, and primary findings remained largely unchanged (Figure 6). Notably, PHQ3 had previously functioned as a key bridge symptom linking sleep disturbances with emotional symptoms. After its removal, PHQ4 (fatigue) assumed this bridging role, connecting emotional symptoms to sleep problems—particularly between daytime dysfunction and depressive symptoms (Figure 7). Thus, while excluding PHQ3 weakened direct sleep–emotion linkages, PHQ4 compensated by maintaining network connectivity, underscoring the intermediary function of fatigue in the emotion–sleep relationship.

## 4 Discussion

This study conducted a cross-sectional survey among Chinese college students enrolled in basketball courses. Through network analysis, a comprehensive network of depressive symptoms, anxiety symptoms, and sleep problems was constructed to identify and evaluate core symptoms and possible interactions among common psychological issues within the context of physical activity. The study revealed that the participating college students generally exhibited psychological problems, with 16.08% presenting anxiety symptoms, 19.83% experiencing depressive symptoms, and 23.34% suffering from sleep disorders, consistent with existing epidemiological survey results (Conteh et al., 2022; Deng et al., 2021; Lin et al., 2025).

The network analysis results for depressive-anxiety symptoms indicated that GAD2 (uncontrollable worry) possessed the highest intensity centrality, followed by GAD6 (irritability) and PHQ8 (psychomotor retardation/agitation), positioning them as key drivers in maintaining or exacerbating the depressive and anxious cycle. These findings align with existing evidence (Milgram and Timpano, 2024) and have also been observed in other populations, such as the elderly and patients with chronic diseases (Zhang et al., 2024; Chen et al., 2023). Uncontrollable worry, a core symptom of generalized anxiety disorder, is defined as excessive and persistent worry that is often disproportionate to actual circumstances. Chinese college students commonly face intense academic competition and future employment pressure, which can readily lead to excessive worry. Limited mental health resources may impede college students' access to timely and effective help, causing worry to accumulate over time and develop into an uncontrollable state. Unhealthy lifestyle behaviors, such as dependence on electronic devices, may also impair college students' capacity to cope with stress. Bridge symptoms, which connect two distinct psychological symptom clusters, can facilitate the development of comorbid psychological symptoms. GAD5 (restlessness), GAD6 (irritability), and GAD7 (fear) were identified as key bridge symptoms with high connectivity strength, serving as critical links between anxiety and depression. GAD6 (irritability) notably demonstrated both high centralization and bridge strength, strongly suggesting its role as a core node in the depression-anxiety pathway. Irritability not only impairs social functioning but may also trigger interpersonal conflicts, thereby exacerbating negative emotional cycles. However, some studies suggest that while irritability signifies psychological disorders in children and adolescents, it does not function as a bridge symptom in adults (Groen et al., 2020). This discrepancy may be attributed to varying manifestations and self-perceptions of the same symptom across different developmental stages.

In the tri-domain symptom network of depression-anxiety-sleep disorders constructed in this study, daytime dysfunction was identified as the most significant symptom through which sleep issues influence anxiety and depressive moods. PSQI7 (daytime dysfunction) exhibited the highest standardized intensity centrality and bridging strength, demonstrating strong negative correlations with GAD5 (restlessness) and PHQ9 (suicidal ideation). This indicates that the severity of daytime dysfunction caused by poor sleep quality (manifested as daytime drowsiness, attention deficits, impaired cognitive function, and mood disturbances) correlates with the severity of an individual's pathological state. In such cases, reduced energy and decreased activity may mask internal restlessness and elevate suicidal ideation risks (Sateia, 2014). Studies have shown that sleep disorders and depression are independently associated with increased suicidal behaviors (Becker et al., 2018), and in this study, PHQ9 (suicidal ideation) also exhibited high intensity centrality and bridging strength. Additionally, PSQI4 (sleep efficiency) and PSQI3 (sleep duration) demonstrated the highest edge strength, suggesting their potential as prioritized intervention targets. These highly correlated local symptoms could provide immediate synergistic benefits while concurrently reducing the activation of other symptom nodes. From a neurobiological perspective, sleep and emotion exhibit a bidirectional coupling relationship. Experimental studies have shown that sleep deprivation impairs top-down regulation between the prefrontal cortex and the amygdala, increasing an individual's sensitivity to negative emotions. Furthermore, chronic sleep disruption is associated with impaired emotion regulation, contributing to the development or persistence of anxiety and depression (Ben Simon et al., 2020; Goldstein and Walker, 2014; Verweij et al., 2014).

In university management, identifying high centralization symptoms may facilitate the early detection of individuals requiring psychological intervention. For instance, those exhibiting prominent GAD2 (uncontrollable worry), GAD6 (irritability), PHQ8 (psychomotor retardation/agitation), PSQI7 (daytime dysfunction), and PSQI1 (sleep quality) symptoms should be closely monitored for potential comorbidities of depression, anxiety, or sleep disorders. Based on network psychology theory, interventions specifically targeting bridging symptoms could weaken the overall strength of psychological networks while blocking cross-symptom and cross-diagnosis transmission pathways—especially when these bridging symptoms also serve as core symptoms. Therefore, GAD6 (irritability) and PSQI7 (daytime dysfunction) should be prioritized in psychological network interventions for college students. The emotional regulation effects of physical exercise are dual: while it reduces anxiety and depression levels and improves sleep quality, high-intensity physical activity may overstimulate the sympathetic nervous system, elevating stress hormones like adrenaline and noradrenaline. This physiological arousal correlates with anxiety states. This study, involving participants in basketball elective courses, revealed that even students with moderate exercise levels still exhibited significant psychological issues. Based on comprehensive network analysis, universities should consider appropriately introducing cognitive behavioral therapy, relaxation therapy, and other interventions to improve college students' nighttime sleep quality and daytime functional impairments while further enhancing their stress management and emotional regulation capabilities (Tadros et al., 2023). The gender comparison test results in this study indicate no significant differences in global network strength or structural differences between genders, which aligns with findings from adolescents, the elderly, and others research (Cai et al., 2022; Fang et al., 2025; Wu and Cai, 2024). This suggests that universities may not need to establish separate intervention programs for different genders.

This study constructed a depression-anxiety-sleep symptom network for Chinese college students through a large-sample survey and enhanced the stability and reliability of the network analysis results using bootstrap tests. The study collected scale information from three different psychological symptom domains to avoid information loss in comorbidity symptoms. To the best of our knowledge, this study is the first to apply network psychometric methods to university students enrolled in a basketball elective, offering novel evidence on the comorbid mechanisms linking depression, anxiety, and sleep disturbances within this physically active population. The findings support several actionable recommendations for university mental health practice. At the intervention level, strategies should prioritize key symptomatic nodes, such as irritability and daytime dysfunction, to disrupt pathways of symptom propagation and promote integrated protocols combining sleep management, emotion regulation, and cognitive-behavioral skills. At the prevention level, these bridging symptoms could be incorporated into early-warning indicators and routine screening systems. Finally, at the curriculum level, stress-management and emotion-regulation training could be embedded into sports electives like basketball, leveraging the synergistic benefits of physical activity and psychological well being.

However, this study still possesses the following limitations. First, while the participants were college students enrolled in basketball elective courses, the study did not account for factors such as exercise frequency, intensity, and duration that influence psychological and sleep patterns. Furthermore, the absence of a control group of students without physical exercise should be addressed in future research. Second, this specific sampling introduces potential selection bias regarding physical activity and motivation, potentially limiting generalizability to the broader student population. Future research should validate these findings in more diverse samples. Third, we acknowledge a potential topological overlap between the sleep item of the PHQ-9 (Item 3) and the PSQI components, as both assess sleep disturbances. While this could theoretically inflate specific edge weights due to semantic redundancy, we chose to retain PHQ-3 to preserve the psychometric integrity of the depression construct and to explicitly model the bridging role of sleep symptoms between the two disorders. Future studies could benefit from applying specific statistical controls or checking network stability when removing overlapping items. Fourthly, the cross-sectional design precludes causal inference; longitudinal data are required to determine directional relationships. Lastly, the use of subjective self-report scales may introduce bias due to participants' potential overestimation or underestimation of symptoms. Finally, as a single-center study, the generalizability of the findings requires further validation considering complex geographical and social environmental factors.

## 5 Conclusion

Based on network analysis of depressive, anxiety, and sleep-related symptoms among Chinese college students enrolled in basketball elective courses, this study identifies uncontrollable worry (GAD2), irritability (GAD6), psychomotor agitation/retardation (PHQ8), and daytime dysfunction (PSQI7) as core and bridge symptoms in the comorbid symptom network. These findings underscore the potential utility of interventions that specifically target such pivotal symptom nodes, offering a theoretical foundation and practical direction for improving mental health within this student population. Given the inherent limitations of cross-sectional designs in establishing causality, future research should incorporate longitudinal approaches to investigate the dynamic evolution of these symptom networks and to evaluate the efficacy of targeted interventions. Such work will be essential for developing more effective strategies to promote mental health among university students.

## Data Availability

The raw data supporting the conclusions of this article will be made available by the authors, without undue reservation.
