# Supplementary material for: Depression, anxiety, and sleep problems among Chinese university students enrolled in basketball elective courses: a network psychometric analysis
Source: Front Psychol. 2026 Mar 4;17:1738600. doi: 10.3389/fpsyg.2026.1738600 (PMC12996064; doi:10.3389/fpsyg.2026.1738600)
Supplement: Supplementary file 1 [file Table_1.docx]

**Supplementary material**


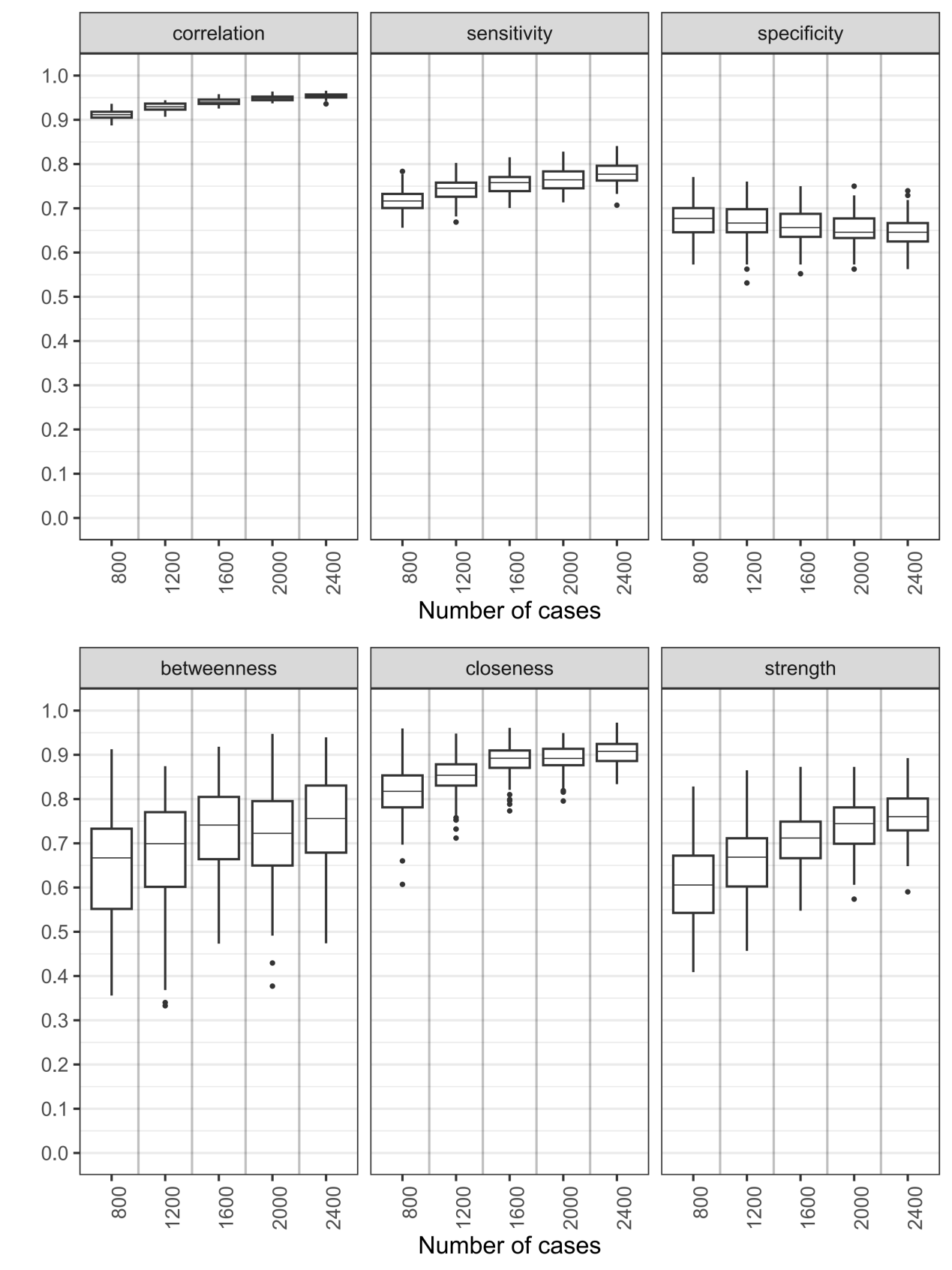


**Figure S1.** Statistical power simulation of the comorbid depression–anxiety–sleep problem symptom network among the study participants. Upper panels depict sensitivity and specificity as functions of case numbers; lower panels illustrate the stability of betweenness, closeness, and strength centrality for bridge symptoms across increasing sample sizes. Dashed lines mark 95 % bootstrapped CIs; the arrow indicates the recommended minimum n ≈ 1 800 required to achieve statistical power ≥ 0.80.


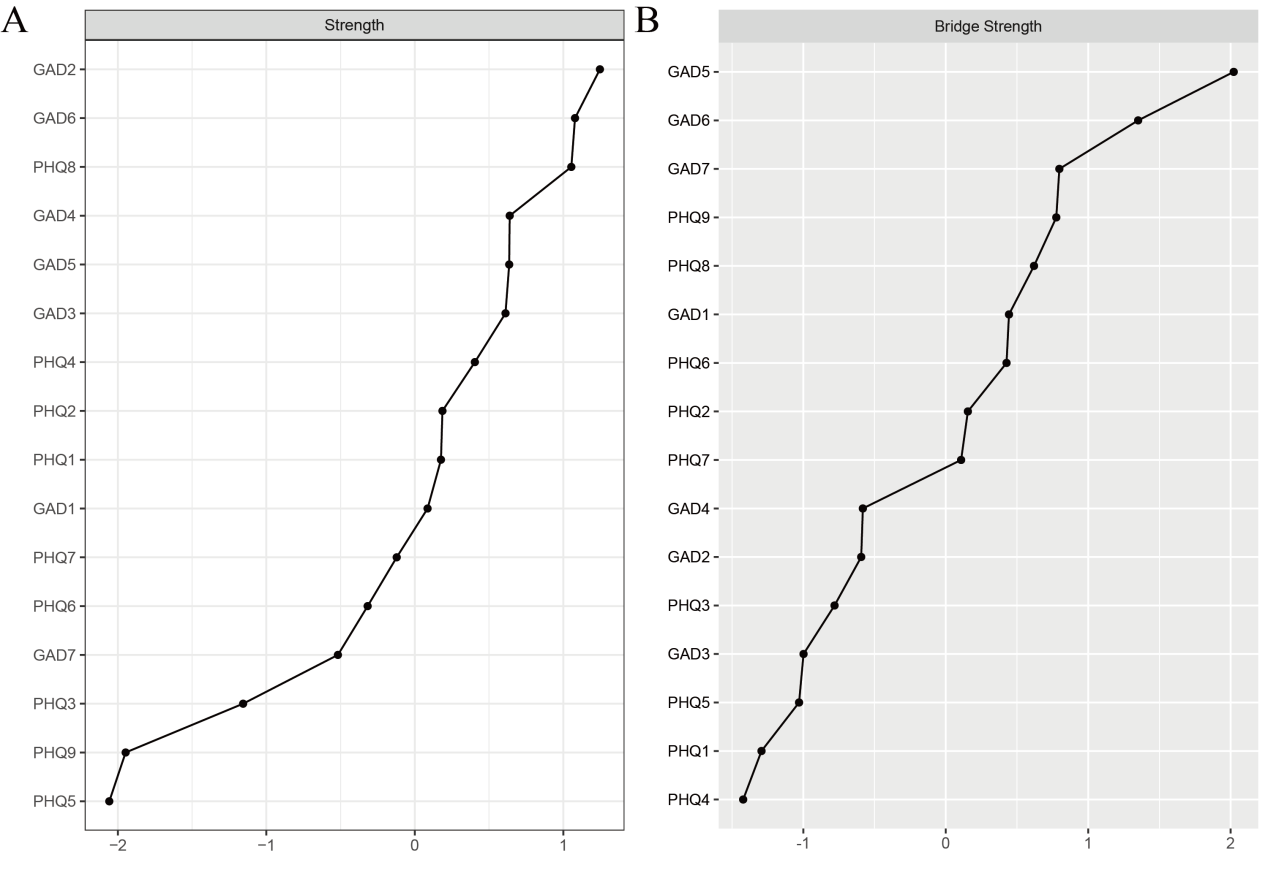


**Figure S2.** Standardized strength and bridge strength centrality of network structure of depression and anxiety symptoms among the study participants (z-scores). (A) strength centrality; (B) bridge strength centrality.


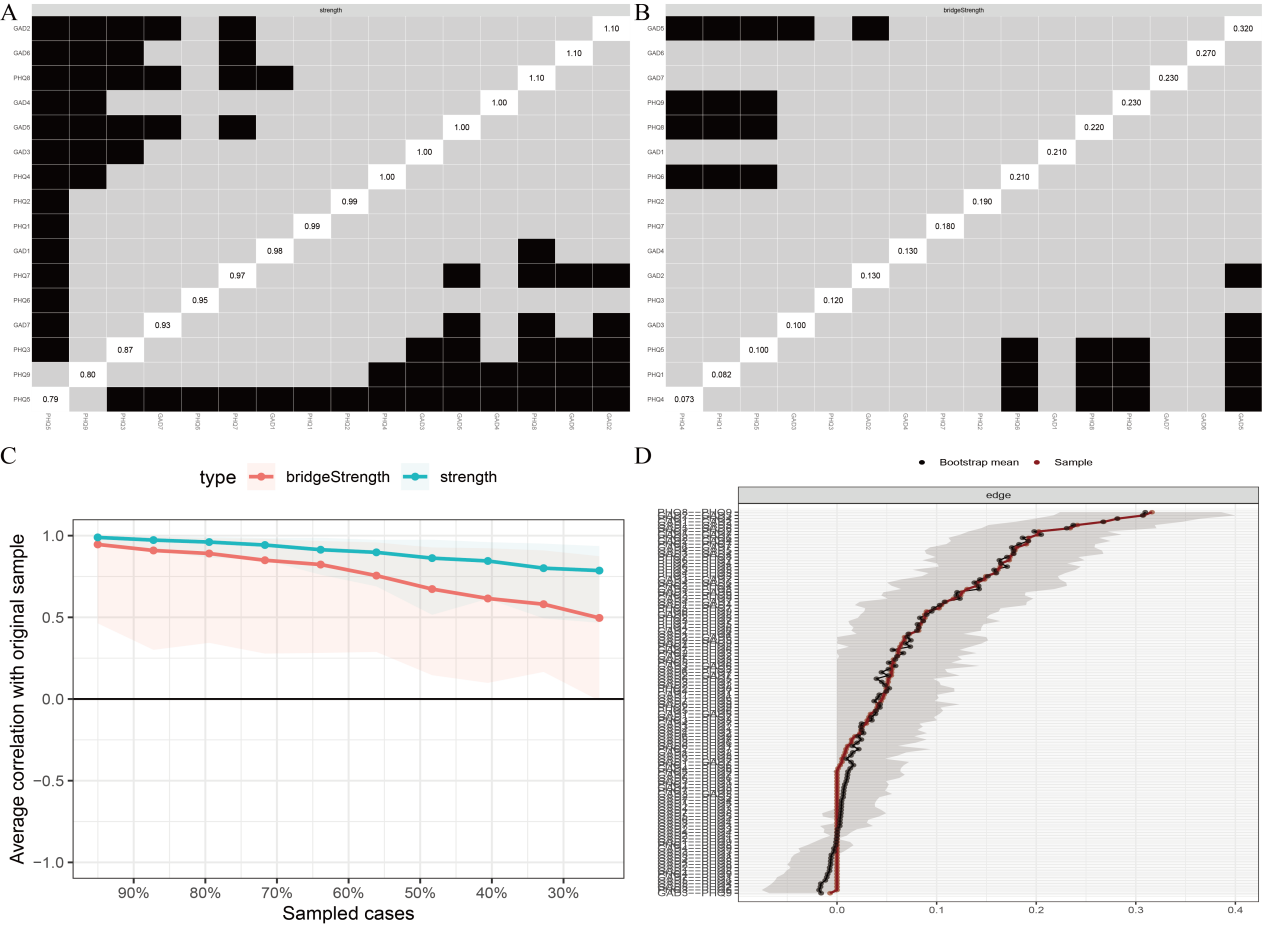


**Figure S3.** Stability and accuracy diagnostics for the depression–anxiety symptom network in the study participants. (A) Bootstrapped difference tests (2,000 draws) for nodal strength centrality. (B) Nonparametric bootstrapped difference test for bridge strength. Gray cells denote non-significant differences (α = 0.05); black cells indicate significant differences; diagonal white cells contain original point estimates. (C) Post-hoc analysis of the stability of node strength and bridge strength. The x-axis represents the proportion of cases excluded (0–0.5); the y-axis shows Pearson correlations between centrality metrics in the original network and those in re-estimated networks. Solid line = strength, dashed line = bridge strength; CS ≥ 0.50 indicates acceptable stability. (D) Bootstrapped 95% confidence intervals for estimated edges. The red lines represent sample estimates; gray bands depict 2,000-bootstrap 95% CIs. Edge identifiers (PHQx–GADy) are aligned along the right-hand y-axis.


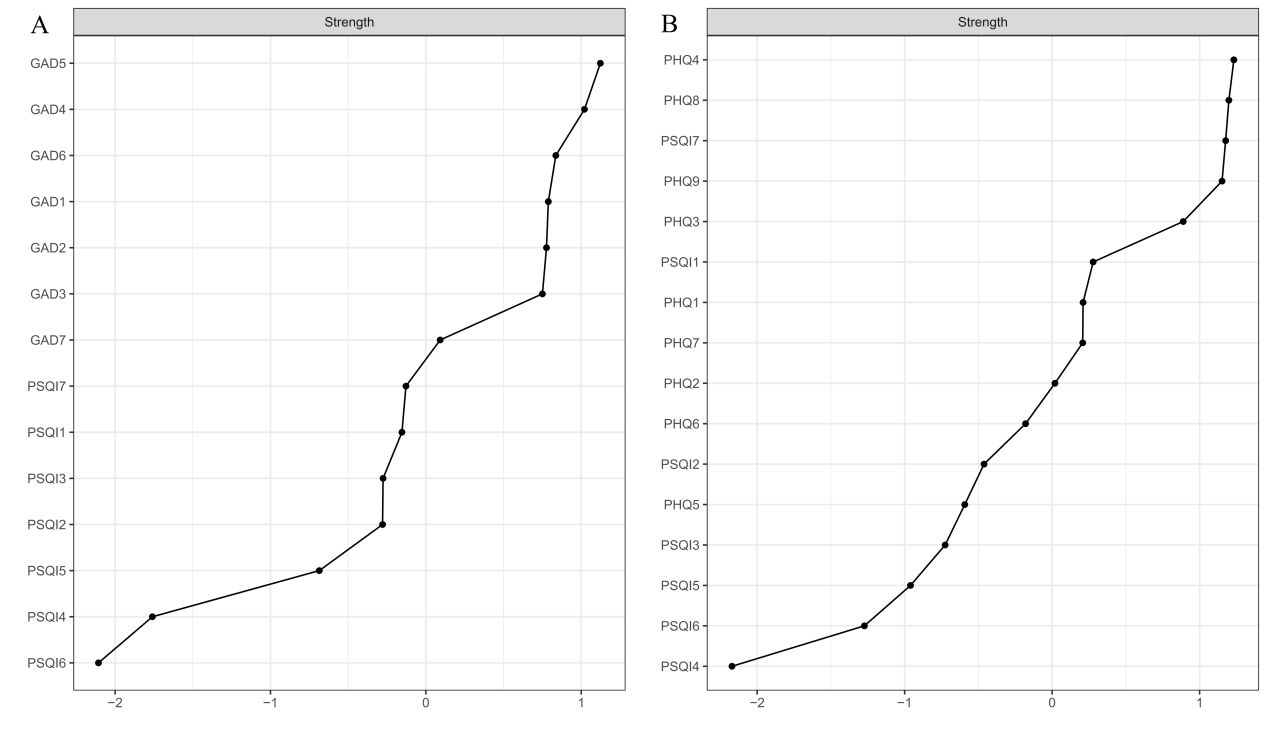


**Figure S4.** Standardized strength centrality plot between sleep problems to symptoms of (A) anxiety and (B) depression (z-scores).


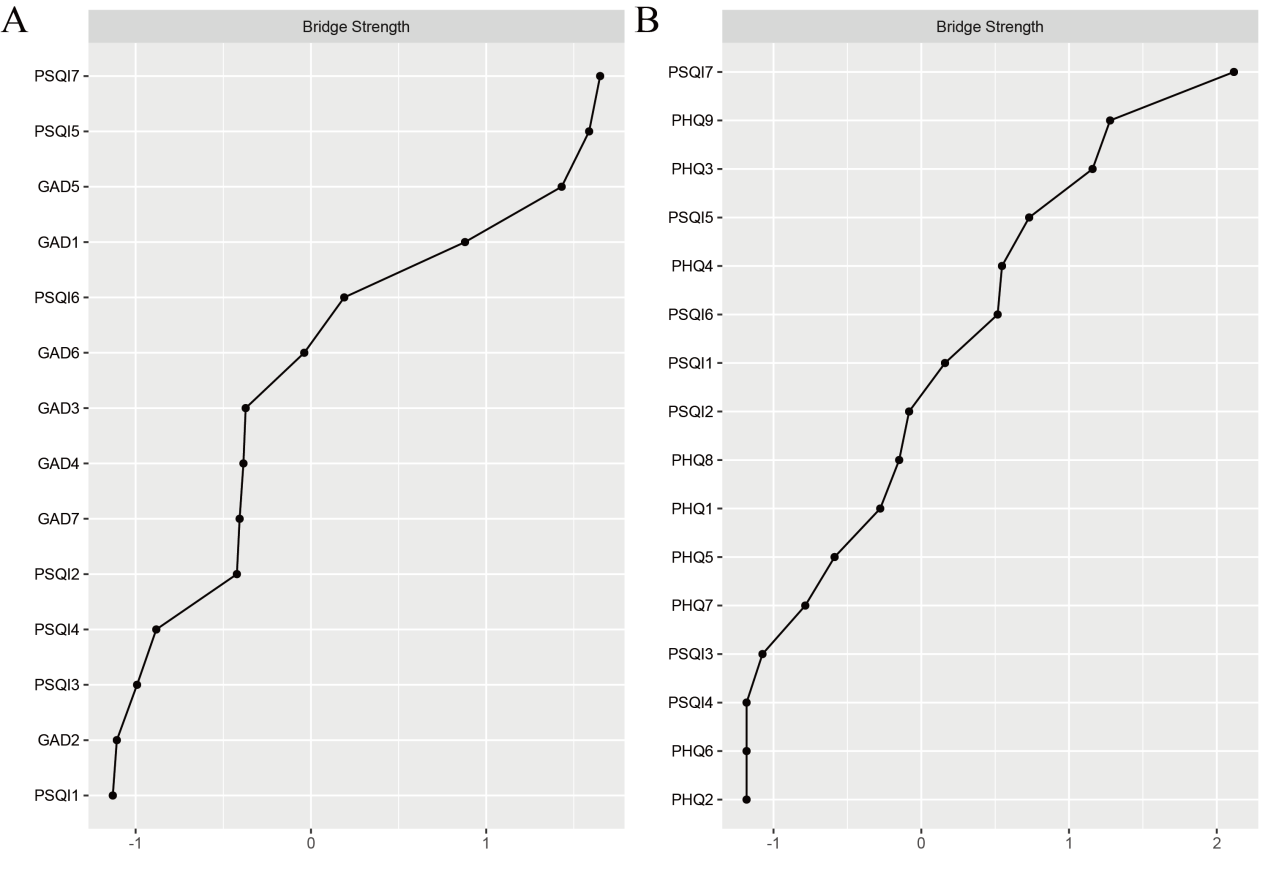


**Figure S5.** Standardized bridge strength centrality plot between sleep problems to symptoms of (A) anxiety and (B) depression (z-scores).


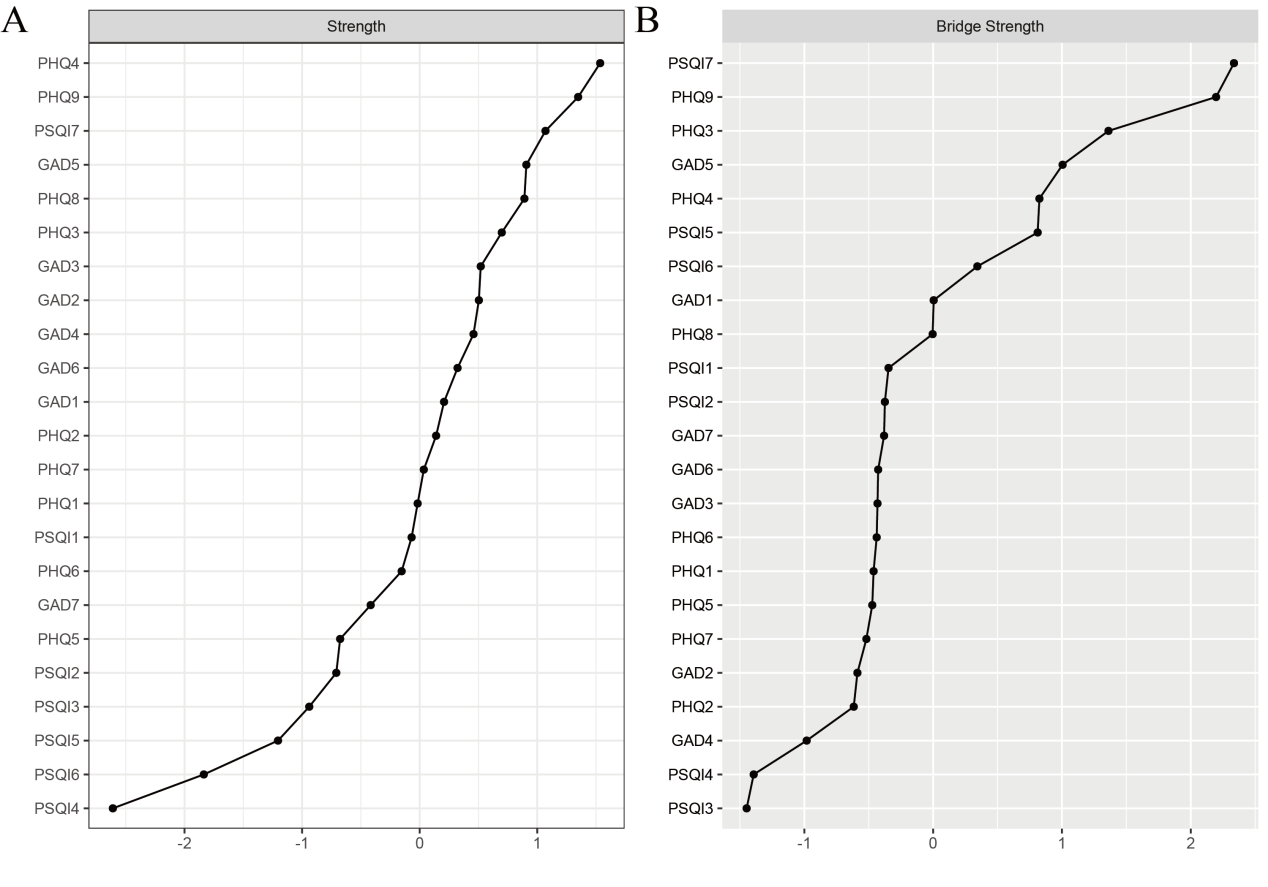


**Figure S6.** Standardized strength and bridge strength centrality of network structure of anxiety, depression and sleep problem symptoms among the study participants (z-scores). (A) strength centrality; (B) bridge strength centrality.


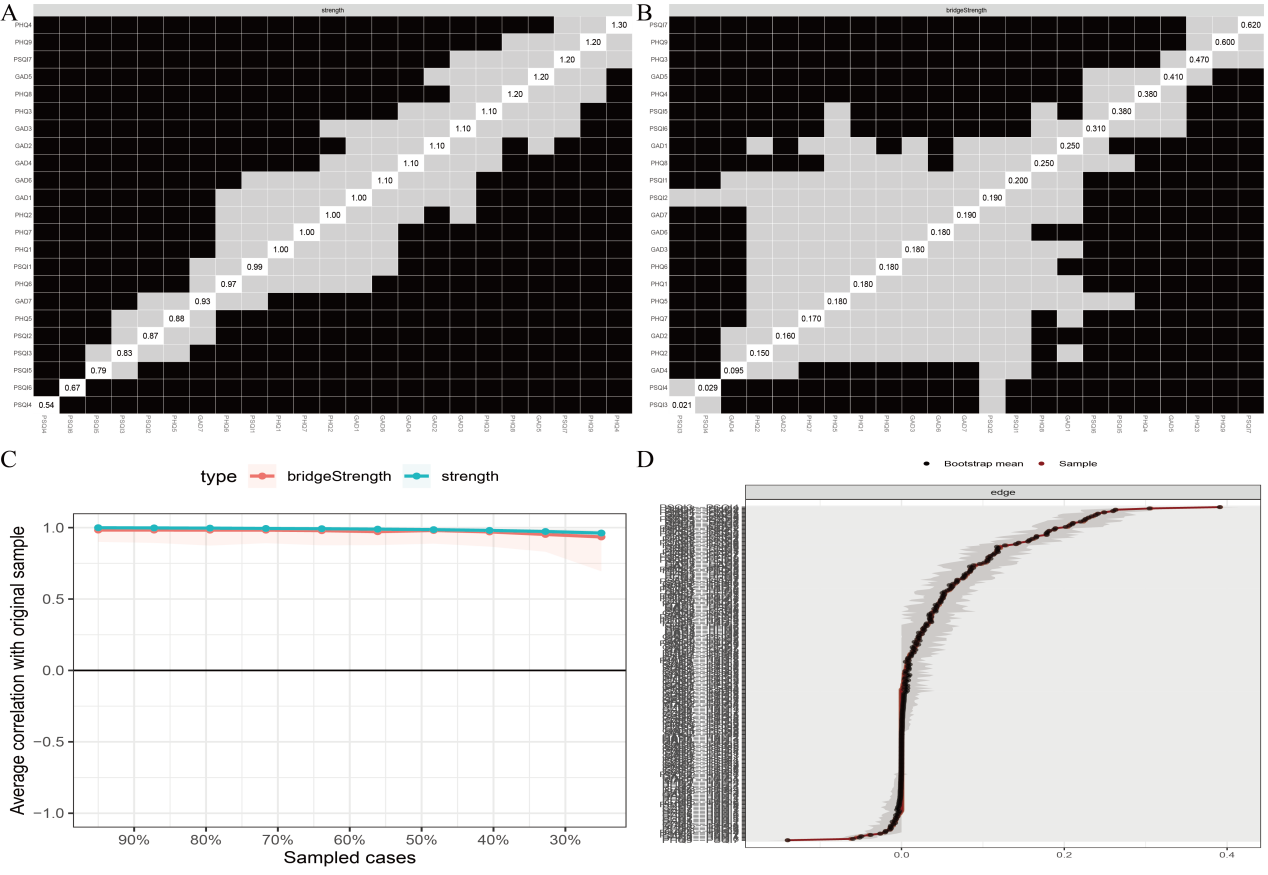


**Figure S7.** Stability and accuracy diagnostics for the anxiety, depression and sleep problem symptoms network in the study participants. (A) Bootstrapped difference tests (2,000 draws) for nodal strength centrality. (B) Nonparametric bootstrapped difference test for bridge strength. Gray cells denote non-significant differences (α = 0.05); black cells indicate significant differences; diagonal white cells contain original point estimates. (C) Post-hoc analysis of the stability of node strength and bridge strength. The x-axis represents the proportion of cases excluded (0–0.5); the y-axis shows Pearson correlations between centrality metrics in the original network and those in re-estimated networks. Solid line = strength, dashed line = bridge strength; CS ≥ 0.50 indicates acceptable stability. (D) Bootstrapped 95% confidence intervals for estimated edges. The red lines represent sample estimates; gray bands depict 2,000-bootstrap 95% CIs. Edge identifiers (PHQx–GADy) are aligned along the right-hand y-axis.

**Table S1.** Item-level mapping of the three key modules: anxiety (GAD-7), depression (PHQ-9) and sleep problems (PSQI) in the study participants.

| **Scale** | **Abbr** | **Symptoms** | **Items** |
| --- | --- | --- | --- |
| **Anxiety** | GAD1 | Nervousness | 1.Feeling nervous, anxious or on edge. |
|  | GAD2 | Uncontrollable Worry | 2.Not being able to stop or control worrying. |
|  | GAD3 | Excessive Worry | 3.Worrying too much about different things. |
|  | GAD4 | Trouble Relaxing | 4.Trouble relaxing. |
|  | GAD5 | Restlessness | 5.Being so restless that it is hard to sit still. |
|  | GAD6 | Irritability | 6.Becoming easily annoyed or irritable. |
|  | GAD7 | Feeling Afraid | 7.Feeling afraid as if something awful might happen. |
| **Depression** | PHQ1 | Anhedonia | 1.I am not interested in doing anything and lack motivation. |
|  | PHQ2 | Sad Mood | 2. Feeling low, depressed, or hopeless. |
|  | PHQ3 | Sleep Dysregulation | 3. Difficulty falling asleep, restless sleep, or excessive sleep. |
|  | PHQ4 | Fatigue | 4. Often feel tired and lack energy. |
|  | PHQ5 | Appetite Changes | 5. Loss of appetite or overeating. |
|  | PHQ6 | Low Self-Evaluation | 6. Feeling bad about oneself, or feeling like a failure, or disappointing oneself or one's family. |
|  | PHQ7 | Concentration Difficulty | 7. Difficulty concentrating, such as being unable to concentrate while reading or watching TV. |
|  | PHQ8 | Psychomotor Retardation/Agitation | 8. Move or speak slowly until others have already noticed. Or on the contrary, more irritable or restless than usual. |
|  | PHQ9 | Suicidal Ideation | 9. There is a thought that it is better to die or harm oneself in some way. |
| **Sleep problem** | PSQI1 | Sleep Quality | 6.In the past month, overall, how do you feel about your sleep quality. |
|  | PSQI2 | Sleep Latency | 2.In the past month, it usually takes ____ minutes from going to bed to falling asleep. 5.In the past month, I have been troubled by the following situations that affect my sleep: a. Difficulty falling asleep (unable to fall asleep within 30 minutes). |
|  | PSQI3 | Sleep Duration | 4.In the past month, I have usually slept for _____ hours per night (not equal to bed rest time). |
|  | PSQI4 | Sleep Efficiency | 1.In the past month, I usually go to bed at ____ o'clock at night. 2.In the past month, it usually takes ____ minutes from going to bed to falling asleep. 4.In the past month, I have usually slept for _____ hours per night (not equal to bed rest time). |
|  | PSQI5 | Sleep Disturbance | 5.In the past month, I have been troubled by the following situations that affect my sleep: b. Easy to wake up at night or wake up early; c. Going to the bathroom at night; d. Difficulty breathing; e. Coughing or snoring loudly; f. Feeling cold; g. Feeling hot; h. Have nightmares; i. Pain and discomfort; j. Other things that affect sleep. |
|  | PSQI6 | Hypnotic Medication | 7.Have you been using medication for hypnosis in the past month? |
|  | PSQI7 | Daytime Dysfunction | 8.Have you often felt tired in the past month? 9.Have you had insufficient energy to do things in the past month? |

**Table S2.** Descriptive statistics, centrality indices and node predictability of anxiety (GAD-7), depression (PHQ-9) and sleep problems (PSQI) in the study participants.

| **Items context** | **Mean** | **SD** | **Skewness** | **Kurtosis** | **Strength** | **Bridge strength** | **Predictability** |
| --- | --- | --- | --- | --- | --- | --- | --- |
| GAD1 | 0.667 | 0.882 | 1.198 | 0.533 | 1.038 | 0.252 | 0.859 |
| GAD2 | 0.611 | 0.863 | 1.297 | 0.762 | 1.091 | 0.158 | 0.881 |
| GAD3 | 0.639 | 0.880 | 1.259 | 0.650 | 1.094 | 0.182 | 0.873 |
| GAD4 | 0.617 | 0.870 | 1.305 | 0.778 | 1.083 | 0.095 | 0.881 |
| GAD5 | 0.542 | 0.842 | 1.491 | 1.315 | 1.163 | 0.411 | 0.860 |
| GAD6 | 0.597 | 0.862 | 1.351 | 0.912 | 1.059 | 0.183 | 0.867 |
| GAD7 | 0.576 | 0.866 | 1.405 | 1.000 | 0.927 | 0.191 | 0.835 |
| PHQ1 | 0.687 | 0.880 | 1.174 | 0.532 | 0.998 | 0.178 | 0.784 |
| PHQ2 | 0.664 | 0.859 | 1.182 | 0.585 | 1.026 | 0.153 | 0.807 |
| PHQ3 | 0.632 | 0.884 | 1.286 | 0.696 | 1.126 | 0.468 | 0.767 |
| PHQ4 | 0.720 | 0.886 | 1.092 | 0.333 | 1.275 | 0.383 | 0.810 |
| PHQ5 | 0.694 | 0.907 | 1.156 | 0.352 | 0.880 | 0.176 | 0.740 |
| PHQ6 | 0.617 | 0.884 | 1.315 | 0.743 | 0.974 | 0.181 | 0.808 |
| PHQ7 | 0.630 | 0.886 | 1.271 | 0.625 | 1.007 | 0.169 | 0.808 |
| PHQ8 | 0.546 | 0.850 | 1.479 | 1.241 | 1.160 | 0.251 | 0.827 |
| PHQ9 | 0.453 | 0.805 | 1.761 | 2.202 | 1.241 | 0.601 | 0.760 |
| PSQI1 | 0.739 | 0.784 | 0.871 | 0.250 | 0.989 | 0.196 | 0.390 |
| PSQI2 | 1.043 | 0.923 | 0.483 | -0.697 | 0.875 | 0.192 | 0.446 |
| PSQI3 | 0.792 | 0.840 | 0.950 | 0.369 | 0.834 | 0.021 | 0.324 |
| PSQI4 | 0.470 | 0.815 | 1.734 | 2.131 | 0.536 | 0.029 | 0.196 |
| PSQI5 | 0.842 | 0.809 | 0.741 | 0.039 | 0.786 | 0.381 | 0.531 |
| PSQI6 | 0.225 | 0.625 | 2.997 | 8.539 | 0.674 | 0.306 | 0.244 |
| PSQI7 | 1.002 | 1.016 | 0.545 | -0.955 | 1.192 | 0.623 | 0.440 |

**Table S3.** Correlation matrix of anxiety (GAD-7), depression (PHQ-9) and sleep problems (PSQI).

|  | **GAD1** | **GAD2** | **GAD3** | **GAD4** | **GAD5** | **GAD6** | **GAD7** | **PHQ1** | **PHQ2** | **PHQ3** | **PHQ4** | **PHQ5** | **PHQ6** | **PHQ7** | **PHQ8** | **PHQ9** | **PSQI1** | **PSQI2** | **PSQI3** | **PSQI4** | **PSQI5** | **PSQI6** | **PSQI7** |
| --- | --- | --- | --- | --- | --- | --- | --- | --- | --- | --- | --- | --- | --- | --- | --- | --- | --- | --- | --- | --- | --- | --- | --- |
| **GAD1** | 0.00 |  |  |  |  |  |  |  |  |  |  |  |  |  |  |  |  |  |  |  |  |  |  |
| **GAD2** | 0.26 | 0.00 |  |  |  |  |  |  |  |  |  |  |  |  |  |  |  |  |  |  |  |  |  |
| **GAD3** | 0.24 | 0.24 | 0.00 |  |  |  |  |  |  |  |  |  |  |  |  |  |  |  |  |  |  |  |  |
| **GAD4** | 0.08 | 0.18 | 0.22 | 0.00 |  |  |  |  |  |  |  |  |  |  |  |  |  |  |  |  |  |  |  |
| **GAD5** | 0.02 | 0.10 | 0.00 | 0.20 | 0.00 |  |  |  |  |  |  |  |  |  |  |  |  |  |  |  |  |  |  |
| **GAD6** | 0.14 | 0.04 | 0.09 | 0.23 | 0.21 | 0.00 |  |  |  |  |  |  |  |  |  |  |  |  |  |  |  |  |  |
| **GAD7** | 0.04 | 0.11 | 0.12 | 0.07 | 0.23 | 0.17 | 0.00 |  |  |  |  |  |  |  |  |  |  |  |  |  |  |  |  |
| **PHQ1** | 0.05 | 0.00 | 0.02 | 0.00 | 0.00 | 0.00 | 0.00 | 0.00 |  |  |  |  |  |  |  |  |  |  |  |  |  |  |  |
| **PHQ2** | 0.04 | 0.03 | 0.03 | 0.01 | 0.00 | 0.00 | 0.02 | 0.21 | 0.00 |  |  |  |  |  |  |  |  |  |  |  |  |  |  |
| **PHQ3** | 0.00 | 0.00 | 0.00 | 0.05 | 0.02 | 0.02 | 0.00 | 0.08 | 0.09 | 0.00 |  |  |  |  |  |  |  |  |  |  |  |  |  |
| **PHQ4** | 0.04 | 0.00 | 0.00 | 0.00 | -0.01 | 0.03 | 0.00 | 0.26 | 0.16 | 0.17 | 0.00 |  |  |  |  |  |  |  |  |  |  |  |  |
| **PHQ5** | 0.01 | 0.00 | 0.02 | 0.00 | 0.00 | 0.04 | 0.00 | 0.11 | 0.05 | 0.12 | 0.18 | 0.00 |  |  |  |  |  |  |  |  |  |  |  |
| **PHQ6** | 0.02 | 0.07 | 0.03 | 0.01 | 0.00 | 0.00 | 0.05 | 0.06 | 0.18 | 0.00 | 0.04 | 0.05 | 0.00 |  |  |  |  |  |  |  |  |  |  |
| **PHQ7** | 0.00 | 0.06 | 0.02 | 0.00 | 0.02 | 0.02 | 0.01 | 0.10 | 0.03 | 0.05 | 0.08 | 0.11 | 0.21 | 0.00 |  |  |  |  |  |  |  |  |  |
| **PHQ8** | 0.00 | 0.00 | 0.00 | 0.00 | 0.12 | 0.04 | 0.00 | 0.00 | 0.04 | 0.08 | 0.01 | 0.08 | 0.16 | 0.24 | 0.00 |  |  |  |  |  |  |  |  |
| **PHQ9** | 0.00 | 0.00 | -0.02 | 0.00 | 0.11 | 0.00 | 0.09 | 0.00 | 0.12 | 0.08 | 0.00 | 0.00 | 0.10 | 0.03 | **0.31** | 0.00 |  |  |  |  |  |  |  |
| **PSQI1** | 0.00 | 0.00 | 0.00 | 0.00 | 0.00 | 0.00 | 0.00 | -0.01 | 0.00 | 0.12 | 0.00 | 0.00 | 0.00 | -0.01 | 0.00 | -0.06 | 0.00 |  |  |  |  |  |  |
| **PSQI2** | 0.01 | 0.00 | 0.00 | 0.00 | 0.00 | 0.00 | 0.00 | 0.00 | 0.00 | 0.16 | 0.00 | 0.00 | 0.00 | 0.00 | -0.01 | -0.01 | 0.19 | 0.00 |  |  |  |  |  |
| **PSQI3** | 0.00 | 0.00 | 0.00 | 0.00 | 0.00 | 0.01 | 0.00 | 0.00 | 0.00 | 0.00 | 0.00 | 0.00 | 0.00 | 0.00 | 0.00 | 0.00 | 0.11 | 0.18 | 0.00 |  |  |  |  |
| **PSQI4** | -0.01 | 0.00 | -0.01 | 0.00 | 0.01 | 0.00 | 0.00 | 0.00 | 0.00 | 0.00 | 0.00 | 0.00 | 0.00 | 0.00 | 0.00 | 0.00 | -0.02 | 0.04 | **0.39** | 0.00 |  |  |  |
| **PSQI5** | 0.02 | 0.00 | 0.00 | 0.00 | 0.00 | 0.02 | 0.01 | 0.03 | 0.01 | 0.08 | 0.05 | 0.06 | 0.00 | 0.00 | 0.04 | 0.05 | 0.08 | 0.23 | 0.01 | 0.00 | 0.00 |  |  |
| **PSQI6** | 0.00 | 0.00 | 0.00 | 0.00 | 0.07 | 0.01 | 0.00 | 0.00 | -0.01 | 0.01 | -0.06 | 0.00 | 0.00 | -0.01 | 0.01 | 0.13 | 0.14 | 0.00 | 0.06 | 0.02 | 0.04 | 0.00 |  |
| **PSQI7** | 0.05 | 0.00 | 0.04 | 0.02 | -0.05 | 0.00 | 0.00 | 0.06 | 0.00 | -0.01 | 0.19 | 0.04 | 0.00 | 0.01 | -0.03 | -0.14 | 0.25 | 0.05 | 0.07 | -0.04 | 0.05 | 0.11 | 0.00 |

**Table S4.** Strength and bridge strength linking sleep problems (PSQI) to anxiety (GAD-7) and depression (PHQ-9).

| **Sleep problems** | **Strength** | | **Bridge Strength** | |
| --- | --- | --- | --- | --- |
|  | **Anxiety** | **Depression** | **Anxiety** | **Depression** |
| **PSQI1** | 0.867 | 1.094 | 0.006 | 0.268 |
| **PSQI2** | 0.841 | 0.925 | 0.069 | 0.219 |
| **PSQI3** | 0.842 | 0.865 | 0.018 | 0.021 |
| **PSQI4** | 0.538 | 0.537 | 0.028 | 0.000 |
| **PSQI5** | 0.758 | 0.812 | 0.247 | 0.381 |
| **PSQI6** | 0.466 | 0.741 | 0.123 | 0.339 |
| **PSQI7** | 0.872 | 1.298 | 0.252 | 0.658 |
